# Supplementary material for: Awareness, knowledge, and beliefs about Human Papillomavirus and its vaccine among Egyptian medical students: A cross-sectional national study
Source: PLoS One. 2025 Dec 5;20(12):e0337411. doi: 10.1371/journal.pone.0337411 (PMC12680150; doi:10.1371/journal.pone.0337411)
Supplement: S1 Table — (PDF) [file pone.0337411.s001.pdf]

S1 Table. Comparison of HPV knowledge between preclinical and clinical medical students.

Characteristic	Clinical N = 1,264	Preclinical N = 236	p-value
HPV is a sexually transmitted infection.	1,175 (98%)	155 (95%)	0.072
HPV is a sexually transmitted infection. (0=False, 1=True)	1,127 (98%)	147 (95%)	0.058
There is a cure for HPV.	771 (64%)	87 (53%)	0.008
There is a cure for HPV. (0=True, 1=False)	408 (35%)	69 (45%)	0.026
Having one type of HPV means you cannot acquire new types.	84 (7%)	16 (9.8%)	0.20
Having one type of HPV means you cannot acquire new types. (0=True, 1=False)	1,072 (93%)	139 (90%)	0.20
There is a screening test commonly used to test males for HPV.	632 (52%)	105 (64%)	0.004
There is a screening test commonly used to test males for HPV. (0=True, 1=False)	554 (48%)	56 (36%)	0.005
An abnormal Pap smear may indicate that a woman has HPV.	1,007 (84%)	137 (84%)	0.90
An abnormal Pap smear may indicate that a woman has HPV. (0=False, 1=True)	967 (84%)	129 (83%)	0.90
Most genital HPV infections do not clear up on their own.	772 (64%)	113 (69%)	0.20
Most genital HPV infections do not clear up on their own. (0=True, 1=False)	419 (36%)	49 (32%)	0.30
A person usually has symptoms when infected with HPV.	371 (31%)	70 (43%)	0.002
A person usually has symptoms when infected with HPV. (0=True, 1=False)	793 (69%)	86 (55%)	<0.001
HPV is not a very common virus.	294 (24%)	36 (22%)	0.50
HPV is not a very common virus. (0=True, 1=False)	873 (76%)	120 (77%)	0.60

HPV infection can cause genital warts.	1,165 (97%)	144 (88%)	<0.001
HPV infection can cause genital warts. (0=False, 1=True)	1,118 (97%)	136 (88%)	<0.001
HPV infection can cause genital herpes.	632 (52%)	104 (64%)	0.007
HPV infection can cause genital herpes. (0=True, 1=False)	548 (47%)	52 (34%)	0.001
Certain types of HPV can lead to cervical cancer in women.	1,158 (96%)	150 (92%)	0.014
Certain types of HPV can lead to cervical cancer in women. (0=False, 1=True)	1,113 (96%)	143 (92%)	0.013
HPV can lay dormant in the body for years without symptoms.	1,047 (87%)	136 (83%)	0.20
HPV can lay dormant in the body for years without symptoms. (0=False, 1=True)	1,002 (87%)	128 (83%)	0.15
A person's chances of getting HPV increase with the number of sexual partners.	1,152 (96%)	144 (88%)	<0.001
A person's chances of getting HPV increase with number of sexual partners. (0=False, 1=True)	1,106 (96%)	136 (88%)	<0.001
Most people with HPV have visible signs or symptoms of infection.	421 (35%)	67 (41%)	0.12
Most people with HPV have visible signs or symptoms of infection. (0=True, 1=False)	753 (65%)	89 (57%)	0.056
Genital warts can cause cervical cancer.	938 (78%)	118 (72%)	0.12
Genital warts can cause cervical cancer. (0=True, 1=False)	252 (22%)	42 (27%)	0.14
Condoms are not effective in preventing HPV.	320 (27%)	71 (44%)	<0.001
Condoms are not effective in preventing HPV. (0=True, 1=False)	851 (74%)	91 (59%)	<0.001
HPV can cause penile cancer.	745 (62%)	119 (73%)	0.006
HPV can cause penile cancer. (0=False, 1=True)	706 (61%)	111 (72%)	0.012

HPV can cause anal cancer.	721 (60%)	116 (71%)	0.006
HPV can cause anal cancer. (0=False, 1=True)	687 (60%)	108 (70%)	0.015
HPV can cause oropharyngeal cancer.	793 (66%)	109 (67%)	0.80
HPV can cause oropharyngeal cancer. (0=False, 1=True)	755 (65%)	101 (65%)	>0.90
Nearly all sexually active people will contract HPV at some point.	643 (53%)	103 (63%)	0.019
Nearly all sexually active people will contract HPV at some point. (0=False, 1=True)	608 (53%)	97 (63%)	0.020
