# Supplementary material for: Awareness, knowledge, and beliefs about Human Papillomavirus and its vaccine among Egyptian medical students: A cross-sectional national study
Source: PLoS One. 2025 Dec 5;20(12):e0337411. doi: 10.1371/journal.pone.0337411 (PMC12680150; doi:10.1371/journal.pone.0337411)
Supplement: S2 Table — (PDF) [file pone.0337411.s002.pdf]

S2 Table. Comparison of attitudes toward HPV and the HPV vaccine between preclinical and clinical medical students.

Attitude Statement	Clinical N = 1,264	Preclinical N = 236	p-value
The HPV vaccination is recommended only for women and girls.	277 (33%)	23 (27%)	0.20
The HPV vaccination is recommended only for women and girls. (0=True, 1=False)	543 (67%)	56 (71%)	0.50
The HPV vaccination is often associated with serious side effects.	185 (22%)	36 (42%)	<0.001
The HPV vaccination is often associated with serious side effects. (0=True, 1=False)	641 (79%)	48 (61%)	<0.001
The HPV vaccine is given as a single shot.	393 (47%)	37 (43%)	0.50
The HPV vaccine is given as a single shot. (0=True, 1=False)	432 (53%)	46 (58%)	0.40
It is best to receive the HPV shot before being sexually active.	737 (88%)	75 (87%)	>0.90
It is best to receive the HPV shot before being sexually active. (0=False, 1=True)	707 (87%)	68 (86%)	0.80
It is too late for teenagers who already had sex to have the HPV vaccine.	145 (17%)	22 (26%)	0.055
It is too late for teenagers who already had sex to have the HPV vaccine. (0=True, 1=False)	670 (83%)	57 (72%)	0.020
If a woman has obtained HPV vaccination, she will not need Pap smears.	134 (16%)	21 (24%)	0.045
If a woman has obtained HPV vaccination, she will not need Pap smears. (0=True, 1=False)	682 (84%)	59 (75%)	0.030
The HPV vaccine will prevent all causes of HPV-associated cancers.	299 (36%)	31 (36%)	>0.90

The HPV vaccine will prevent all causes of HPV-associated cancers. (0=True, 1=False)	527 (65%)	53 (67%)	0.70
HPV vaccine is offered only to sexually active people.	223 (27%)	19 (22%)	0.40
HPV vaccine is offered only to sexually active people. (0=True, 1=False)	593 (73%)	60 (76%)	0.60
The HPV vaccine will protect from getting HPV-associated cancers.	p = 0.30		
Agree	457 (38%)	57 (35%)	
Disagree	77 (6.4%)	11 (6.7%)	
Not Sure	378 (31%)	47 (29%)	
Strongly Agree	152 (13%)	19 (12%)	
Strongly Disagree	140 (12%)	29 (18%)	
The HPV vaccine will be effective in preventing HPV infection.	p = 0.13		
Agree	484 (40%)	72 (44%)	
Disagree	107 (8.9%)	23 (14%)	
Not Sure	294 (24%)	32 (20%)	
Strongly Agree	179 (15%)	20 (12%)	
Strongly Disagree	140 (12%)	16 (9.8%)	
Getting an HPV vaccine will benefit my health.	p = 0.40		
Agree	460 (38%)	59 (36%)	
Disagree	90 (7.5%)	17 (10%)	
Not Sure	279 (23%)	44 (27%)	
Strongly Agree	213 (18%)	27 (17%)	
Strongly Disagree	162 (13%)	16 (9.8%)	
If I have an HPV infection, it would be disruptive to my health.	p = 0.041		
Agree	383 (32%)	59 (36%)	
Disagree	200 (17%)	18 (11%)	

Not Sure	339 (28%)	45 (28%)	
Strongly Agree	129 (11%)	27 (17%)	
Strongly Disagree	153 (13%)	14 (8.6%)	
If I have HPV-associated cancer, it would threaten my relationship with my partner.	p = 0.033		
Agree	385 (32%)	60 (37%)	
Disagree	99 (8.2%)	23 (14%)	
Not Sure	289 (24%)	28 (17%)	
Strongly Agree	262 (22%)	34 (21%)	
Strongly Disagree	169 (14%)	18 (11%)	
HPV-associated cancer is a life-threatening disease.	p = 0.90		
Agree	389 (32%)	49 (30%)	
Disagree	135 (11%)	21 (13%)	
Not Sure	282 (23%)	43 (26%)	
Strongly Agree	236 (20%)	30 (18%)	
Strongly Disagree	162 (13%)	20 (12%)	
I think the HPV vaccine is unsafe.	p = 0.50		
Agree	67 (5.6%)	6 (3.7%)	
Disagree	476 (40%)	74 (45%)	
Not Sure	409 (34%)	56 (34%)	
Strongly Agree	15 (1.2%)	1 (0.6%)	
Strongly Disagree	237 (20%)	26 (16%)	
I feel embarrassed to get an HPV vaccine because it is for an STI.	p = 0.004		
Agree	169 (14%)	25 (15%)	
Disagree	370 (31%)	35 (21%)	
Not Sure	334 (28%)	45 (28%)	
Strongly Agree	35 (2.9%)	13 (8%)	
Strongly Disagree	296 (25%)	45 (28%)	

It is hard to find a provider or clinic that has the vaccine.	p = 0.60		
Agree	250 (21%)	29 (18%)	
Disagree	199 (17%)	34 (21%)	
Not Sure	559 (46%)	77 (47%)	
Strongly Agree	62 (5.1%)	8 (4.9%)	
Strongly Disagree	134 (11%)	15 (9.2%)	
I am concerned that the HPV vaccine costs more than I or my parents can pay.	p = 0.60		
Agree	167 (14%)	26 (16%)	
Disagree	277 (23%)	34 (21%)	
Not Sure	578 (48%)	79 (48%)	
Strongly Agree	37 (3.1%)	2 (1.2%)	
Strongly Disagree	145 (12%)	22 (13%)	
I am at risk of contracting HPV.	p = 0.008		
Agree	96 (8%)	17 (10%)	
Disagree	414 (34%)	57 (35%)	
Not Sure	345 (29%)	28 (17%)	
Strongly Agree	18 (1.5%)	1 (0.6%)	
Strongly Disagree	331 (27%)	60 (37%)	
I am at risk for getting cervical cancer.	p > 0.90		
Agree	80 (6.6%)	13 (8%)	
Disagree	360 (30%)	48 (29%)	
Not Sure	314 (26%)	41 (25%)	
Strongly Agree	18 (1.5%)	3 (1.8%)	
Strongly Disagree	432 (36%)	58 (36%)	
