# Supplementary material for: Awareness, knowledge, and beliefs about Human Papillomavirus and its vaccine among Egyptian medical students: A cross-sectional national study
Source: PLoS One. 2025 Dec 5;20(12):e0337411. doi: 10.1371/journal.pone.0337411 (PMC12680150; doi:10.1371/journal.pone.0337411)
Supplement: S3 Table — (PDF) [file pone.0337411.s003.pdf]

S3 Table. Comparison of HPV-related awareness and vaccination practices between preclinical and clinical medical students.

**Practice Item**	**Clinical N = 1,264**	**Preclinical N = 236**	**p-value**
Have you heard about HPV vaccine?	841 (70%)	86 (53%)	<0.001
Have you heard about HPV vaccine? (1=Yes, 0=No)	810 (70%)	79 (51%)	<0.001
Have you received any type of vaccination?	997 (83%)	132 (81%)	0.60
Have you received any type of vaccination? (1=Yes, 0=No)	953 (83%)	124 (80%)	0.40
