# Supplementary material for: Awareness, knowledge, and beliefs about Human Papillomavirus and its vaccine among Egyptian medical students: A cross-sectional national study
Source: PLoS One. 2025 Dec 5;20(12):e0337411. doi: 10.1371/journal.pone.0337411 (PMC12680150; doi:10.1371/journal.pone.0337411)
Supplement: Attachment — Submitted filename: Response to reviewers.pdf [file pone.0337411.s006.pdf]

Academic editor comments

Comment:

Reviewer 2 raises a concern regarding the high similarity with a recently published study, Abdelaziz, M.N., Hefnawy, A., Azzam, H. et al. Knowledge and attitude among Egyptian medical students regarding the role of human papillomavirus vaccine in prevention of oropharyngeal cancer: a questionnaire-based observational study. Sci Rep 15, 3767 (2025). https://doi.org/10.1038/s41598-025-86853-8". Please highlight and comment on any differences with the above-cited study in the discussion section.

Response:

We appreciate the reviewer's observation regarding potential similarity with the recently published study by Abdelaziz et al. (2025). While both studies examine HPV-related knowledge and attitudes among Egyptian medical students, our work differs in several key aspects. First, Abdelaziz et al. focused primarily on awareness of the *role of HPV vaccination in preventing oropharyngeal cancer*, while our study investigates *overall HPV awareness, knowledge, and beliefs*—including its association with multiple cancers, perceived risk, and behavioral intentions toward vaccination. Second, our survey instrument and analytical framework were distinct; we employed a more comprehensive set of variables assessing knowledge domains, vaccine hesitancy, and sociodemographic predictors using multivariable analysis. Third, our study sampled a broader range of universities across Egypt and was conducted during a different time period, thereby providing an independent dataset that complements, rather than duplicates, prior work.

Importantly, the two studies together provide convergent evidence highlighting the persistent gap in HPV-related knowledge among future Egyptian healthcare providers. Our findings extend the literature by identifying contextual factors influencing awareness and vaccination intent, offering additional insight useful for educational and public health interventions in Egypt.

Response to Reviewers

We sincerely thank all reviewers for their thorough evaluation, constructive comments, and valuable suggestions that have strengthened the quality and clarity of our manuscript. Below we provide detailed, point-by-point responses and describe all revisions made.

Reviewer #1

General Comment:
This manuscript proposes an in-depth analysis of HPV awareness among Egyptian medical students. The paper is well written and discusses the importance of targeted interventions in increasing HPV knowledge among medical students. I suggest English language editing and more attention to captions and tables.

Response:
We appreciate the reviewer's positive feedback. The manuscript has undergone comprehensive English language editing, and all tables and captions have been reviewed for clarity, consistency, and formatting.

Comment 1:
Pag. 9. Men are also significantly affected… Please consider replacing the word "strains" with "genotypes".

Response:
We thank the reviewer for the suggestion. The word **"strains"** has been replaced with **"genotypes"** in the revised text.

Comment 2:
It seems that your introduction is too short. Please emphasize more topics, such as HPV prevalence and vaccines' impact on the population. I invite the authors to consider the following works…

Response:
We have expanded the **Introduction** to include global HPV prevalence data and a more detailed discussion of vaccine impact, including references to the suggested works and other recent studies. The new paragraph has been added after the second paragraph of the Introduction.

Revised text:

Globally, HPV is the most common sexually transmitted infection; nearly all sexually active individuals acquire it during their lifetime, with point prevalence of high-risk types around 11–12% among women and any-type genital HPV detected in roughly 45% of men and about one quarter carrying high-risk genotypes. HPV causes almost all cervical cancers and a substantial proportion of anal, vulvar, penile, and oropharyngeal cancers, with cervical cancer alone responsible for approximately 660,000 new cases and 350,000 deaths annually, the majority in low- and middle-income countries. Oral HPV infection is relatively common—national surveys report an overall oral HPV prevalence near 7% in adults, with higher rates in men, HPV16 detected in about 1%, and strong associations with number of oral sex partners and tobacco exposure. Randomized and real-world data indicate that prophylactic vaccination reduces vaccine-type oral HPV, supporting expectations that widespread vaccination will eventually lower HPV-related oropharyngeal cancer burden.

Relevant references have been added as recommended.

Comment 3:
RESULTS: Page 16, TABLE 2 — Please consider specifying that in the items column, there are only the questions and not the correct answers.

Response:
We appreciate this observation. The caption of Table 2 has been updated to clarify that the "Items" column lists **survey questions** rather than correct responses.

Reviewer #2

General comment:

The manuscript analyses HPV awareness among 1,431 medical students in Egypt… The study shows and highlights some important misconceptions… It is well-written and appropriately designed.

Response:

We thank the reviewer for their positive assessment and supportive feedback.

Comment:

I would also like to suggest some papers specifically regarding oral HPV infection and HPV vaccines…

Response:

We have incorporated and cited the suggested references in the **Background** section to strengthen discussion of oral HPV infection and vaccine impact.

Comment:

However, I need to raise an issue regarding the novelty of the study… discuss the differences between your paper and Abdelaziz et al. (2025).

Response:

We appreciate the reviewer's concern regarding overlap. The following paragraph has been added to the **Discussion** section to clarify the distinction between our study and Abdelaziz et al. (2025):

A recently published study by Abdelaziz et al. (2025) examined Egyptian medical students' knowledge regarding HPV and oropharyngeal cancer. Although both studies address HPV awareness among medical students, our work differs in scope and objectives. The present study assesses a larger and more geographically diverse sample from multiple medical faculties, evaluates broader domains of HPV knowledge, attitudes, and preventive practices, and compares awareness across academic levels to identify educational gaps. In contrast, Abdelaziz et al.

focused specifically on knowledge related to HPV-associated oropharyngeal cancer. Our findings therefore complement, rather than duplicate, existing evidence.

Reviewer #3

Major comment 1:
Comparing by education would probably provide more insight… comparison should be made across the educational levels or academic years.

Response:
We thank the reviewer for this insightful suggestion. To address it, we conducted an additional comparison by academic level (preclinical vs. clinical years). The results are presented in three new supplementary tables:

- **Supplementary Table 1:** Knowledge about HPV by academic level
- **Supplementary Table 2:** Attitudes toward HPV and HPV vaccine by academic level
- **Supplementary Table 3:** Practices and awareness by academic level

Summary of findings added to the Results section:

Comparison across educational levels showed that clinical-year students demonstrated significantly higher knowledge on several items, including HPV's causal link to cervical, penile, and anal cancers, and greater awareness that condoms provide partial protection ($p < 0.001$ for several items). However, misconceptions persisted among both groups regarding HPV symptoms and curability. Attitudinal differences were modest; clinical students were less likely to perceive the vaccine as unsafe ($p < 0.001$) and more likely to reject myths such as the vaccine being only for women. Awareness of the HPV vaccine was also significantly higher among clinical students (70% vs. 53%, $p < 0.001$). These analyses reinforce the conclusion that educational exposure influences awareness but cannot confirm causality.

Minor comment 2:
Clarify statement about preventive screening and reference 11.

Response:

Revised sentence in the **Background**:

Preventive screening for HPV-related cancers is also gender-skewed, with well-established cervical cancer screening protocols for women, whereas no validated or routinely implemented screening method exists for HPV-associated cancers in cisgender men. In addition, barriers persist for sexual and gender minority individuals assigned female at birth, as noted previously [15].

Minor comment 3:

Clarify "Students who declined participation were excluded from the analysis."

Response:

This has been corrected in the **Methods** section:

"Students who declined participation were not enrolled in the study."

Minor comment 4:

Page 21: no need of elaboration here—human papillomavirus (HPV).

Response:

The redundant elaboration has been removed from that section.

Minor comment 5:

Revise limitation statement about causality.

Response:

We revised the first paragraph of the **Limitations** section to clarify the causal limitation:

The cross-sectional design captures associations at a single point in time and therefore cannot determine whether increased educational exposure actually leads to greater HPV awareness or more favorable attitudes. While differences across academic years were observed, these reflect

correlations rather than causal effects, as longitudinal data would be required to confirm directional change.

Minor comment 6:
How do the calculated proportions be 100% for Religious Affiliation data in both HPV Awareness category in Table 1?

Response:
Percentages were calculated column-wise within each awareness category, and the sample was religiously homogeneous. To clarify, we added a note beneath Table 1:

"Values are presented as frequencies and column percentages. Percentages for religious affiliation total 100% within each awareness category, reflecting the homogeneity of the sample."

Minor comment 7–9 (Formatting and spelling):
We have standardized all table fonts and sizes to match the manuscript text and corrected the typographical error "Strongly Disgree" to "Strongly Disagree."

Reviewer #4

Comment:
In the methods section, source of awareness by responders was not captured and in the abstract section, the results provided showed the narrative with no statistical values to support the claims made in the findings.

Response:
We appreciate this feedback.

1. **Regarding the source of awareness:** We acknowledge that the questionnaire did not capture how participants first learned about HPV (e.g., through the curriculum, peers, or media). This has now been explicitly clarified in the "Data collection tool" subsection of the Methods as follows:

"The questionnaire did not include an item assessing the source of HPV awareness (e.g., academic curriculum, peers, or media exposure)."

2. The **Abstract** has been updated to include key statistical values (e.g., proportions and p-values) to support the summarized findings.
