# Supplementary material for: Awareness, knowledge, and beliefs about Human Papillomavirus and its vaccine among Egyptian medical students: A cross-sectional national study
Source: PLoS One. 2025 Dec 5;20(12):e0337411. doi: 10.1371/journal.pone.0337411 (PMC12680150; doi:10.1371/journal.pone.0337411)
Supplement: Attachment — Submitted filename: Comments_PONE-D-25-45022.docx [file pone.0337411.s004.docx]

**Overall impression:** The research paper is well written, especially the methodology is clear and easy to understand. Apart from missing page numbers and line numbers, I have one major comment and a few minor comments, and I am referring to the page numbers of the PDF provided-

**Major comment:**

1. Comparing by education would probably provide more insight into factors shaping HPV knowledge and HPV vaccination knowledge, while the sex-based comparison is still useful but more limited, especially for table 2 & 3. Since the author highlighted the importance of “…..*educational interventions, particularly those targeting early academic years, are essential to address these gaps”* in the abstract, comparison should be made across the educational levels or academic years.

**Minor comments:**

2. In your reference no 11, they addressed the limitation of preventive screening for SGM AFAB people, but the author exemplified no preventive screening facility for men (page 9, last sentence of the 2^nd^ paragraph). This needs to be clarified or corrected in the manuscript.
3. The author stated in the methods//Participants and eligibility criteria: “Students who declined participation were excluded from the analysis”. If the students who declined participation, there supposed to be no data at all from them. Or if any students participated, provided data and then withdrawn from the study- in that case, those data would be excluded from analysis. Or, did the author mean-Students who declined participation were excluded from enrollment? This needs to be clarified in the manuscript.
4. Page 21: no need of elaboration here- human papillomavirus (HPV)
5. Page 23: In Limitation section: causality assessment between **increasing** educational exposure and **changing** HPV awareness or attitudes is not possible in cross sectional study, but in my opinion, it was assessed in the current study. In another sense, the author’s statement is not wrong, but better to convey why causality is limited, because cross-sectional data can’t track whether more exposure actually leads to **changes** in awareness or attitudes. The statement can be revised by the author.
6. How do the calculated proportions be 100% for Religious Affiliation data in both HPV Awareness category in table1?
7. The font size of the table titles should be same as manuscript text font size.
8. Spelling needs to be corrected- “Strongly Disgree” in table 5.
9. The font styles for all the tables should be consistent.
